# Locomotor Behaviour of *Blattella germanica* Modified by DEET

**DOI:** 10.1371/journal.pone.0083433

**Published:** 2013-12-23

**Authors:** Valeria Sfara, Gastón A. Mougabure-Cueto, Eduardo N. Zerba, Raúl A. Alzogaray

**Affiliations:** 1 Centro de Investigaciones de Plagas e Insecticidas (CIPEIN-UNIDEF-CITEDEF-CONICET), Villa Martelli, Buenos Aires, Argentina; 2 Instituto de Investigación e Ingeniería Ambiental (3IA), Universidad Nacional de San Martín, San Martín, Buenos Aires, Argentina; Federal University of Viçosa, Brazil

## Abstract

N,N-diethyl-3-methylbenzamide (DEET) is the active principle of most insect repellents used worldwide. However, its toxicity on insects has not been widely studied. The aim of this work is to study the effects of DEET on the locomotor activity of *Blattella germanica*. DEET has a dose-dependent repellent activity on *B. germanica*. Locomotor activity was significantly lower when insects were pre-exposed to 700 µg/cm^2^ of DEET for 20 or 30 minutes, but it did not change when pre-exposure was shorter. Locomotor activity of insects that were pre-exposed to 2.000 µg/cm^2^ of DEET for 10 minutes was significantly lower than the movement registered in controls. No differences were observed when insects were pre-exposed to lower concentrations of DEET. A 30-minute pre-exposure to 700 µg/cm^2^ of DEET caused a significant decrease in locomotor activity. Movement was totally recovered 24 h later. The locomotor activity measured during the exposure to different concentrations of DEET remained unchanged. Insects with decreased locomotor activity were repelled to the same extent than control insects by the same concentration of DEET. We demonstrated that the repellency and modification of locomotor activity elicited by DEET are non-associated phenomena. We also suggested that the reduction in locomotor activity indicates toxicity of DEET, probably to insect nervous system.

## Introduction

An insect repellent has been defined as a chemical substance acting in the steam phase and producing oriented movements of insects away from its source [1], [2]. N,N-diethyl-3-methylbenzamide (DEET) is the active principle of most insect repellents used worldwide. The repellent properties of DEET were discovered in 1946. Ten years later, it came onto the market becoming a successful product due to its effectiveness, persistence and low human toxicity [3]. The repellent effect of DEET has been proved in several blood-sucking and non-blood-sucking insect species [4].

Behavioural and electrophysiological studies showed that DEET acts as a sensory stimulus and can be detected by insect antennae interacting with olfactory as well as gustatory receptors [5]–[15]. These interactions are probably the physiological basis of the repellency elicited by DEET. Recently it was demonstrated that DEET inhibits insect acetylcholinesterase (AChE) [16], suggesting that this compound has a toxic effect on insect nervous system. The biological function of AChE is to terminate the nerve signal transmission through the hydrolysis of the neurotransmitter acetylcholine (Ach). The inhibition of AChE results in the accumulation of Ach in the synaptic cleft, thus producing a continuous stimulation of the post-synaptic neuron, finally causing the disruption of the transmission of the nerve impulse [17]. However, the toxicity of DEET in insects has not been extensively studied. Moreover, it is not clear if there is a mechanistic relationship between the inhibition of the AChE and the repellency phenomenon [16], [18], [19]. Sfara et al. [20] observed an increase in locomotor activity in the blood-sucking bug *Rhodnius prolixus* after these insects were exposed to high concentrations of DEET. Increase in locomotor activity (i.e., hyperactivity) and lack of locomotor activity (i.e., prostration and paralysis) in insects are considered symptoms of poisoning with neurotoxic substances such as pyrethroids [21]–[24]. The interaction of some substances targeted at the nervous system produces a series of well-described symptoms before killing the insect. For example, poisoning with pyrethroids first increases insect locomotor activity, which may be due to the effect of this insecticide in the thoracic ganglia and nerves that control the legs of the animal. As the concentration of the insecticide in the target site increases, the intoxication progresses and symptoms such as tremors, paralysis and death are observed [25], [26]. Gammon et al. [21] recorded the electrical activity of the nerve cord of the cockroach *Periplaneta americana* treated with the pyrethroid d-allethrin as they observed the symptoms of the intoxication. They recorded different electrical activities of the nerve associated to each symptom.

The German cockroach is a worldwide household pest of significance to human health. It was found that these insects transmit several pathogens that affect humans, such as poliomyelitis and hepatitis viruses. The presence of bacteria in the tarsi of cockroaches was also observed. These animals also produce inhaled and ingested allergens [27].

The aim of this work is to determine possible toxicity of DEET by studying the effect of this substance on the locomotor activity of the German cockroach *Blattella germanica*.

## Materials and Methods

### Biological material

In this work 7 to 20 day-old colony-reared adult males of *B. germanica* were used. They were kept at laboratory in an environmental chamber (25 °C and 12:12 h L:D photoperiod). Water and rat pellet were offered to insects *ad libitum*.

### Chemicals

N, N-diethyl-3-methylbenzamide (DEET) 97% pure was from Aldrich (Milwaukee, WI, USA). Acetone was from Merck (Darmstadt, Germany).

### Experimental device

**Figure 1:**
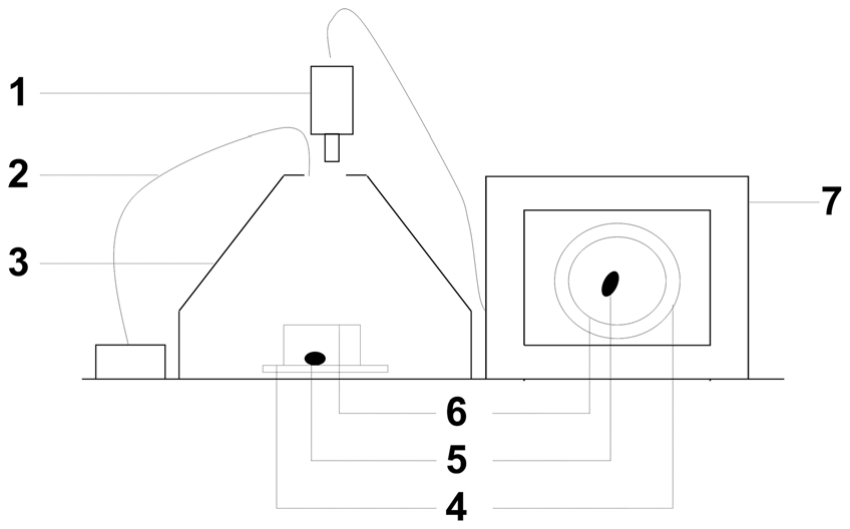
Experimental arena used to determine the spatial distribution of the insects. 1. video camera; 2. optic fibre of light source (halogen lamp 10,500 lux); 3. dark chamber; 4. filter paper; 5. insect; 6. glass ring; 7. monitor.

Figure 1 shows the device used in bioassays. The experimental arenas where locomotor activity and repellency were determined, were isolated with a dark photographic cone. Insect movements were observed using a digital video camera connected to a monitor (Sony, Tokyo, Japan). The arena was illuminated using a halogen white light (Philips 6423 FO, 15V/15W) with an IR filter and a 60 cm optic fibre placed 10 cm above the arena. The illumination level was 10,500 lux. It was measured with a digital lux meter (TES Digital Lux Meter 1330A) at different points of the experimental arena to ensure even illumination of the entire surface.

### Pre-exposure to DEET

Continuous exposure to DEET was achieved using a circular plastic container (diameter: 9 cm). A circular filter paper of the same diameter was treated with 0.5 ml of DEET in acetone. After solvent evaporation, the filter paper was placed on the floor of the container and one insect was put on it. The container was then closed.

### Quantification of locomotor activity

A circular arena (diameter: 11 cm) was divided into eight equal sections. A glass ring (high: 4 cm; diameter: 11 cm) was used to prevent insects from leaving the arena. A male of *B. germanica* was gently placed on the arena. Locomotor activity was quantified by counting the number of crossings to each area of the arena that insects made during 300 seconds.

### Bioassays

Four experimental series were performed to study the effect of DEET on insect locomotor activity. In the first experimental series, one insect was pre-exposed to a filter paper treated with 0.5 ml of a solution of 100 mg/ml of DEET in acetone (700 µg/cm^2^). Different insects were exposed for 1, 10, 20 or 30 minutes. Locomotor activity was quantified immediately after interrupting pre-exposure. Two controls were included in each replicate: (a) one insect pre-exposed to acetone alone, (b) one insect pre-exposed to a non-treated filter paper.

In the second experimental series, one insect was pre-exposed for 10 minutes to a filter paper treated with solutions of DEET in acetone. Different insects were pre-exposed to 350, 700 or 2000 µg/cm^2^ of DEET. Locomotor activity was quantified immediately after interrupting pre-exposure. The two controls described above were included.

In the third experimental series, one insect was pre-exposed during 30 minutes to a filter paper treated with 0.5 ml of a solution of 100 mg/ml of DEET in acetone (700 µg/cm^2^ of DEET). Locomotor activity was quantified 5, 10, 18 and 24 h after interrupting pre-exposure. The two controls described above were included.

In the fourth experimental series, locomotor activity was determined for 300 seconds during exposure to an arena treated with different concentrations of DEET (70, 700 or 2000 µg/cm^2^). Control insects were exposed to an arena treated with acetone only.

In all cases, at least ten independent replicates were performed.

### Quantification of repellency

A circular piece of Whatman No. 1 filter paper (Whatman International Ltd., Miadstone, UK) (diameter: 11 cm) was cut into halves (Zone I and Zone II). Zone I was treated with 0.35 ml of acetone, whereas Zone II was treated with 0.35 ml of a solution of DEET in acetone. The following concentrations were tested: 3.5; 70; 700 or 2,000 µg/cm^2^. After acetone evaporation, both filter paper halves were fitted together and located on the test arena floor. A glass ring (high: 4.5 cm; diameter: 9 cm) was used to prevent the insect from leaving the filter paper. One adult male of *B. germanica* was gently placed in the centre of the filter paper and the time spent by the insect in each zone was monitored during 300 seconds using a digital video camera connected to a monitor (Sony, Tokyo, Japan).

The results were expressed as a Repellency Coefficient [RC  =  (Total Experimental Time – Time in Zone II)/Total Experimental Time]. RC values vary between 0 (maximum attraction) and 1 (maximum repellency). RC  =  0.5 indicates that the insect spent the same time in both zones (random distribution). As controls, one insect was located in an arena where both halves were treated with acetone only. At least ten independent replicates were performed for each bioassay.

### Quantification of repellency after continuous exposure to DEET

A circle of filter paper was treated with 0.5 ml of a 100 mg/ml solution of DEET in acetone (700 µg/cm^2^ of DEET). The filter paper was located at the bottom of a plastic container, and one insect was gently placed on it. The container was closed. After 30 minutes, the insect was moved to an arena divided into halves (a half treated with 350 µg/cm^2^ of DEET; the other half, with acetone alone). At least ten independent replicates were performed.

### Statistical analysis

The RC and the number of crossings in the locomotor activity experiments were statistically analyzed using Kruskall-Wallis test, followed by Dunn’s non-parametric *post-hoc* comparisons test when required.

## Results

**Figure 2:**
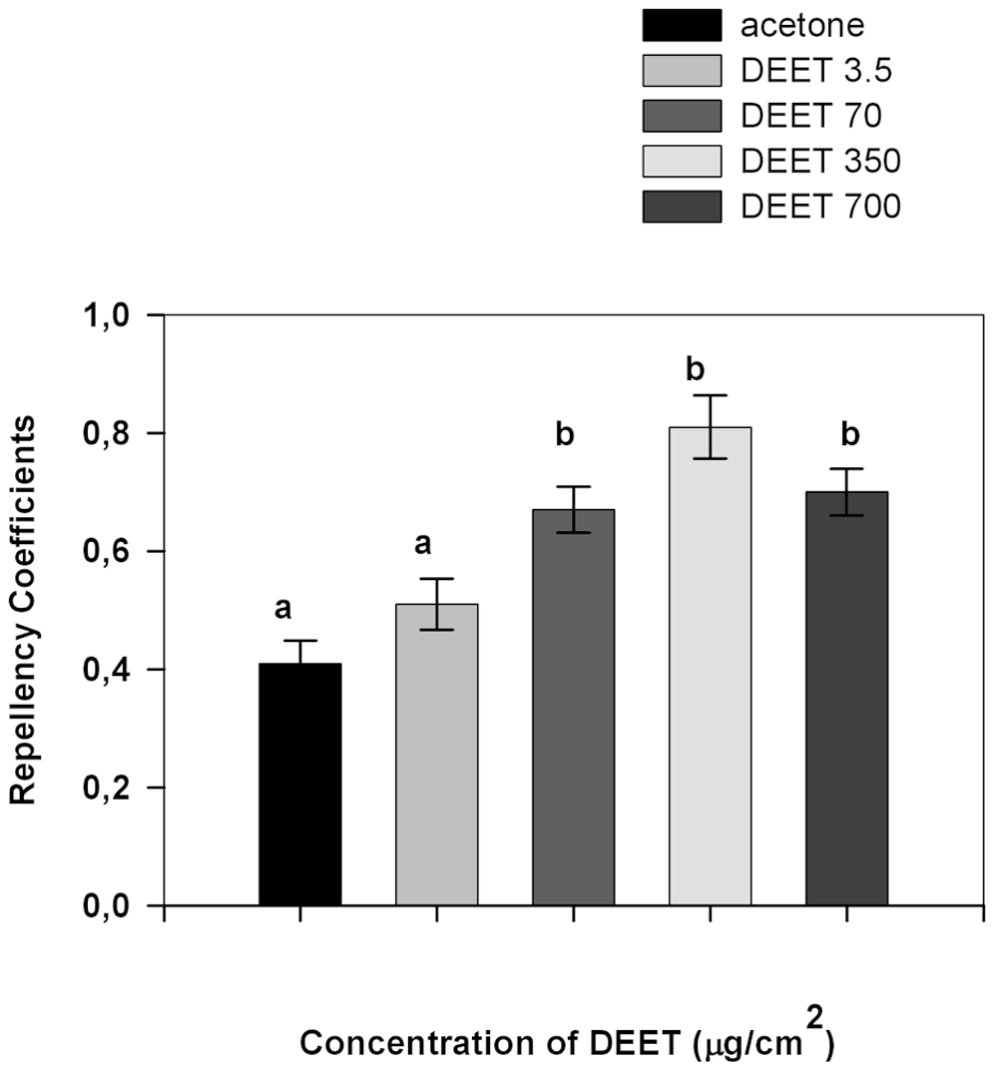
Repellency Coefficient (RC) values calculated for different concentrations of DEET. Dotted line indicates random distribution of the insects (RC  =  0.5). Error bars indicate standard error. Different letters indicate significant differences (p<0.05 Kruskall-Wallis test and Dunńs test for post-hoc comparisons).

The repellency caused by different concentrations of DEET was determined in cockroaches. Figure 2 shows the RC values for different concentrations of DEET in cockroaches exposed to the repellent. Repellency was higher as concentration of DEET increased.

**Figure 3:**
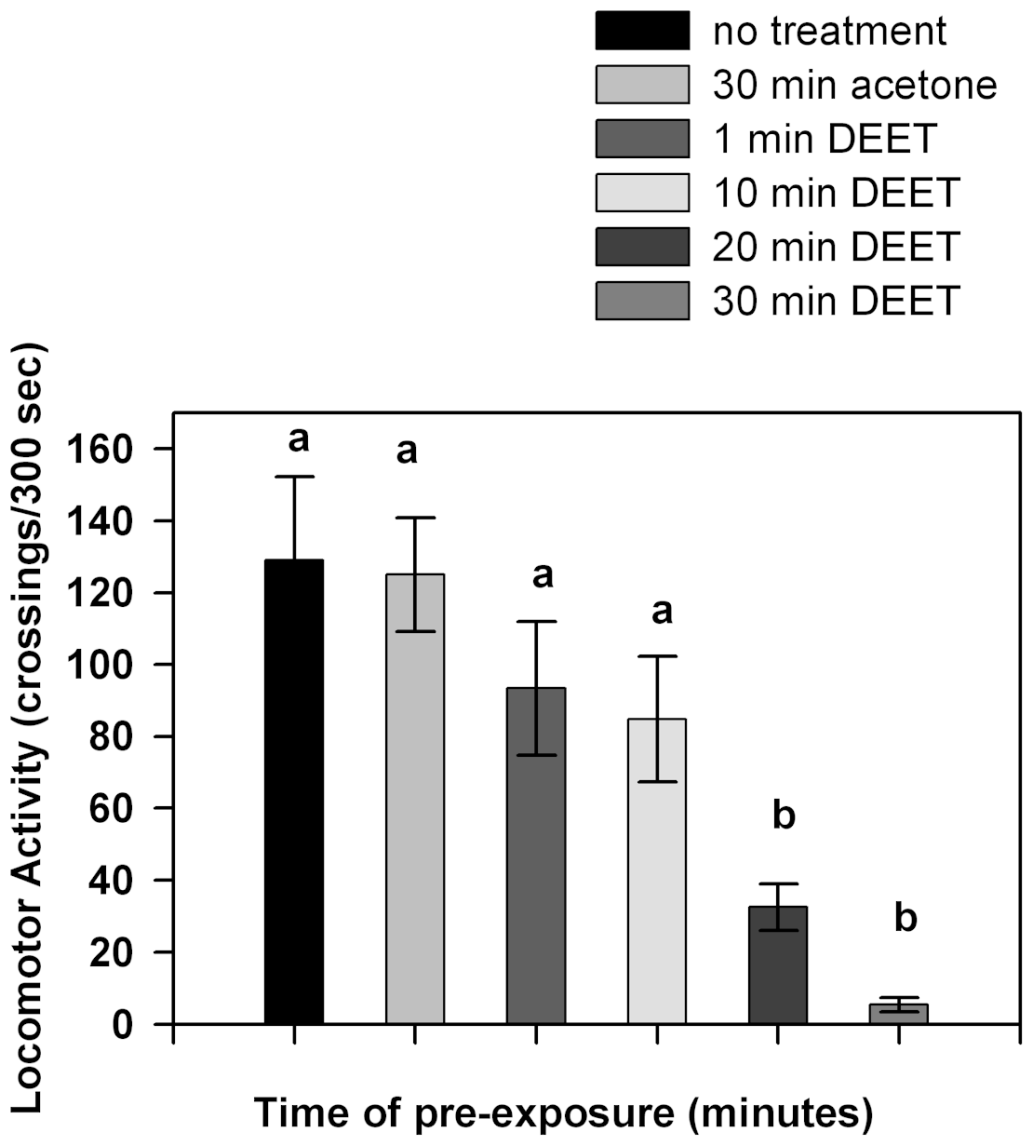
Locomotor activity of insects pre-exposed for different periods to 700 µg/cm^2^ of DEET. Bars represent the mean number of crossings to each area during the experimental time. Error bars indicate standard error. Different letters indicate significant differences (p<0.05 Kruskall-Wallis test and Dunńs test for post-hoc comparisons).

The results of the first experimental series in which insects were pre-exposed to 700 µg/cm^2^ of DEET during different periods are shown in figure 3. The locomotor activity of insects pre-exposed to DEET for 1 or 10 minutes showed no changes compared to control insects (p>0.05). Locomotor activity was significantly lower when insects were pre-exposed to DEET for 20 or 30 minutes (p<0.05).

**Figure 4:**
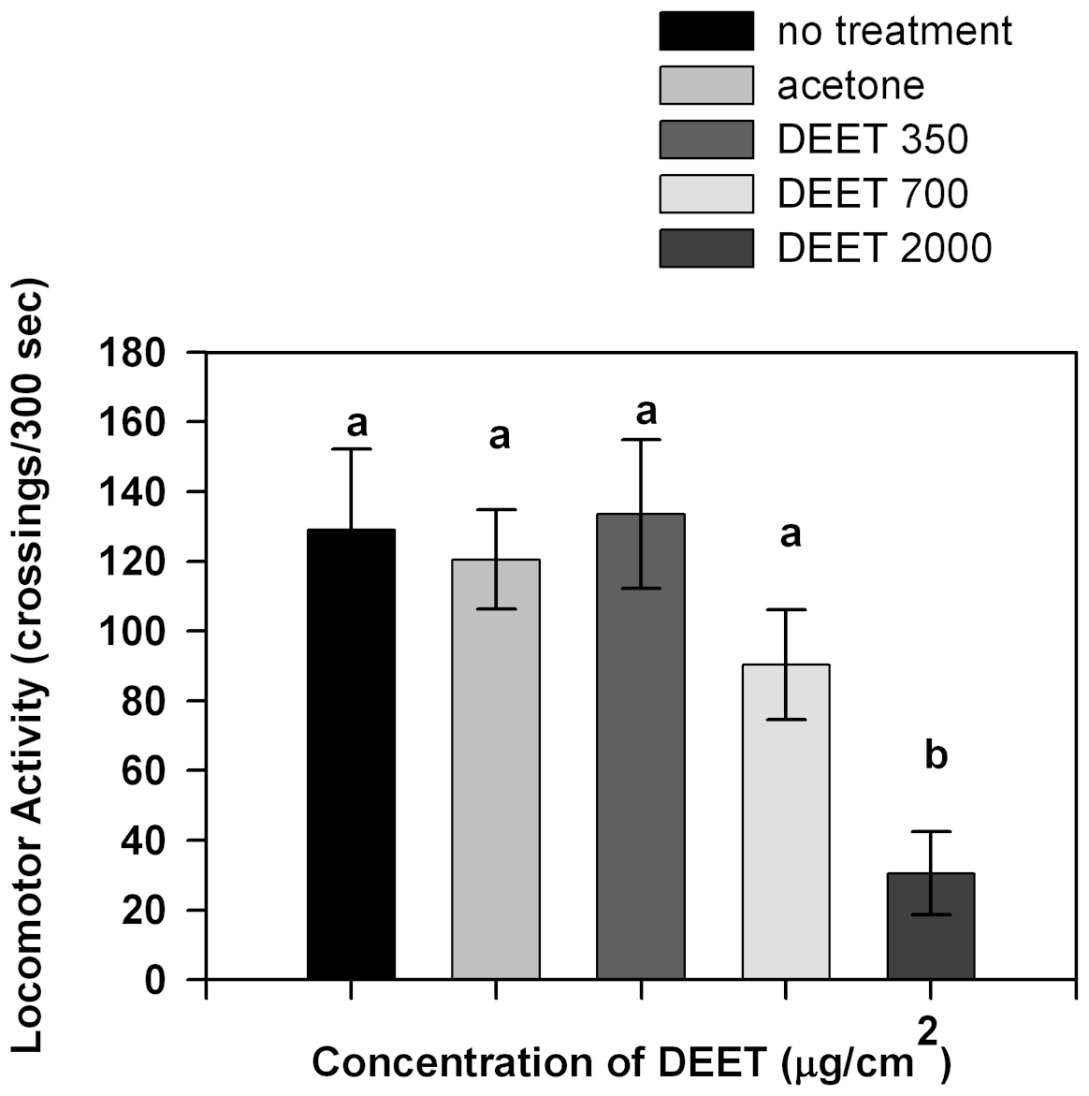
Locomotor activity of insects pre-exposed for 10 minutes to different concentrations of DEET. Bars indicate the mean number of crossings to each area during the experimental time. Error bars indicate standard error. Different letters indicate significant differences (p<0.05 Kruskall-Wallis test and Dunńs test for post-hoc comparisons).

Results obtained from the second experimental series are shown in figure 4. Insects were pre-exposed for 10 minutes to different concentrations of DEET. No differences in locomotor activity were observed when insects were pre-exposed to 3.5 or 700 µg/cm^2^ of DEET for 10 minutes compared to controls (p>0.05). Locomotor activity of insects pre-exposed to 2000 µg/cm^2^ of DEET was significantly lower than the locomotor activity registered in controls (p<0.05).

**Figure 5:**
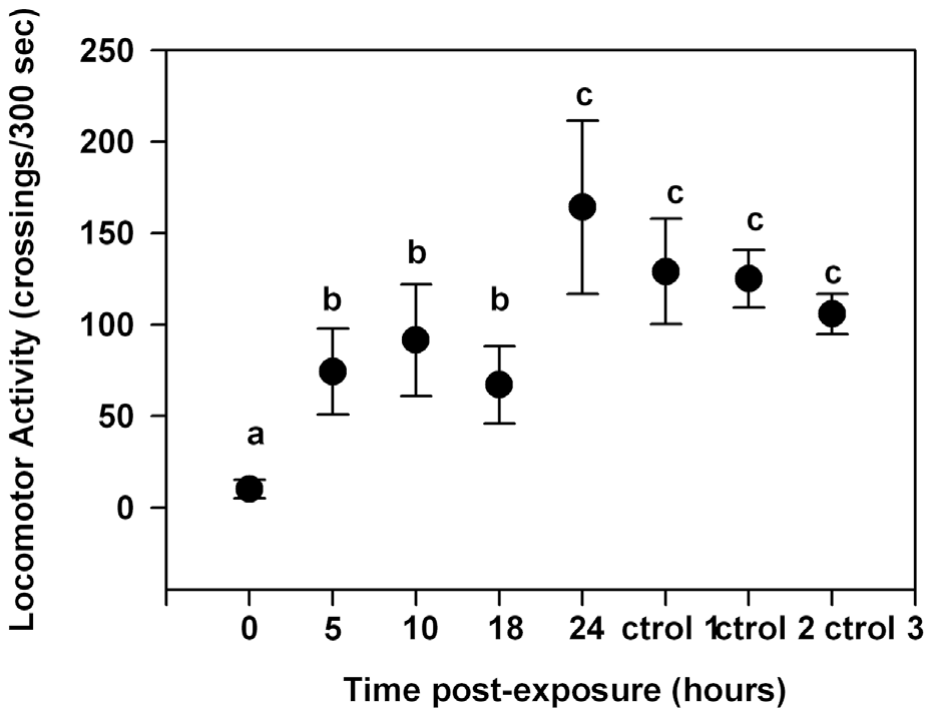
Locomotor activity of insects pre-exposed for 30 minutes to 700 µg/cm^2^ of DEET measured at different periods after pre-exposure. Control 1 is the locomotor activity of non-treated insects; control 2 is the locomotor activity of insects pre-exposed for 30 minutes to acetone and measured immediately after pre-exposure; control 3 is the locomotor activity of insects pre-exposed for 30 minutes to acetone and measured 24 h after pre-exposure. Each point indicates the mean number of crossings to each area during the experimental time. Error bars indicate standard error. Different letters indicate significant differences (p<0.05 Kruskall-Wallis test and Dunńs test for post-hoc comparisons).

Figure 5 shows the locomotor activity of cockroaches pre-exposed to 700 µg/cm^2^ of DEET during 30 minutes, measured at different periods after pre-exposure (third experimental series). Pre-exposure to DEET caused a significant decrease in locomotor activity at period 0 (p<0.05). The locomotor activity was recovered in a period-dependent manner; 24 h after pre-exposure, locomotor activity was not significantly different from controls (p>0.05).

**Figure 6:**
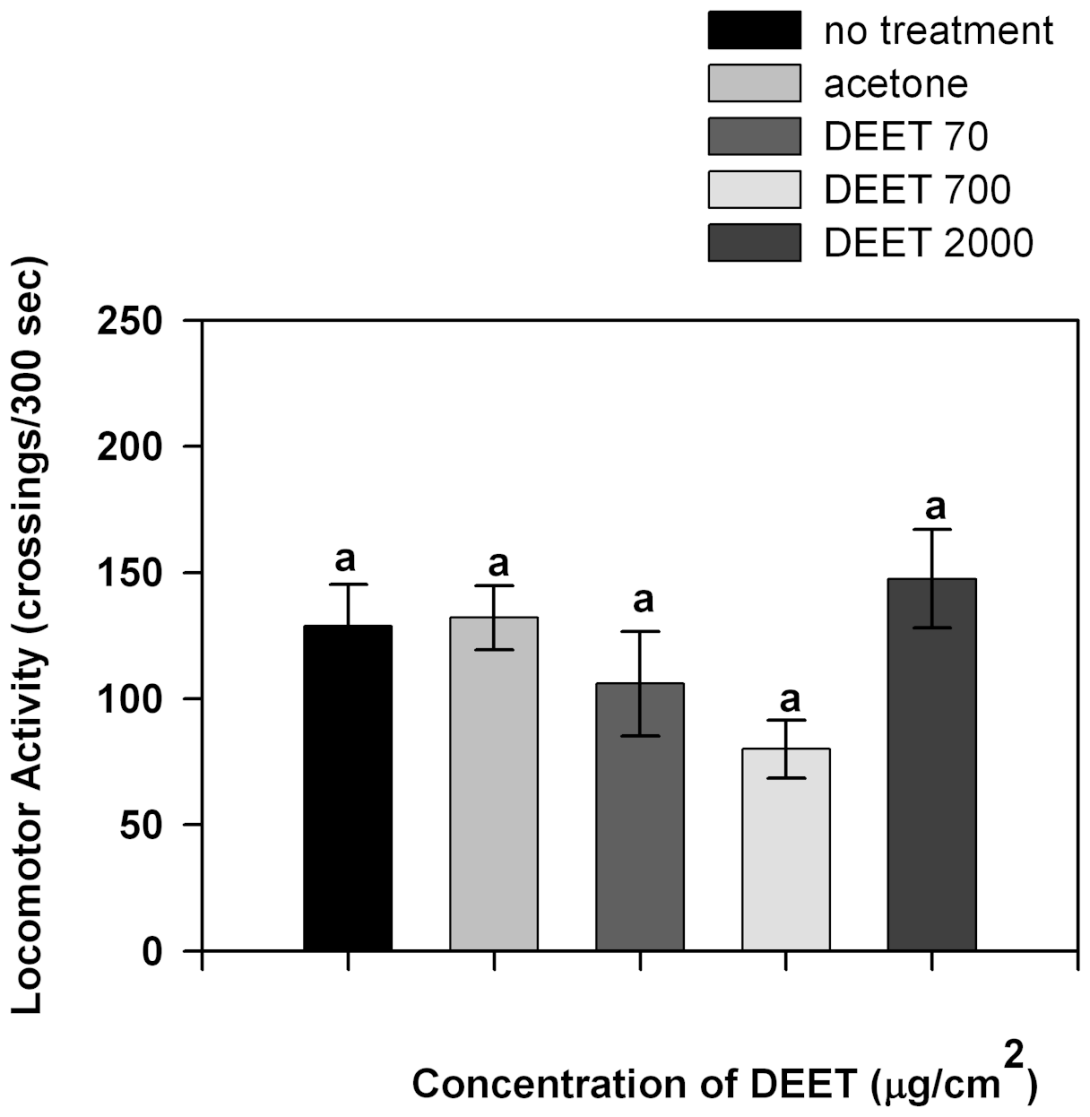
Locomotor activity of insects exposed to different concentrations of DEET measured during the exposure. Bars indicate the mean number of crossings to each area during the experimental time. Error bars indicate standard error. Equal letters indicate no significant differences (p>0.05 Kruskall-Wallis test).

Figure 6 shows the results of the fourth experimental series where the locomotor activity of cockroaches was measured during exposure to different concentrations of DEET. No significant differences were observed in locomotor activity for any studied concentration of DEET (p>0.05).

**Figure 7:**
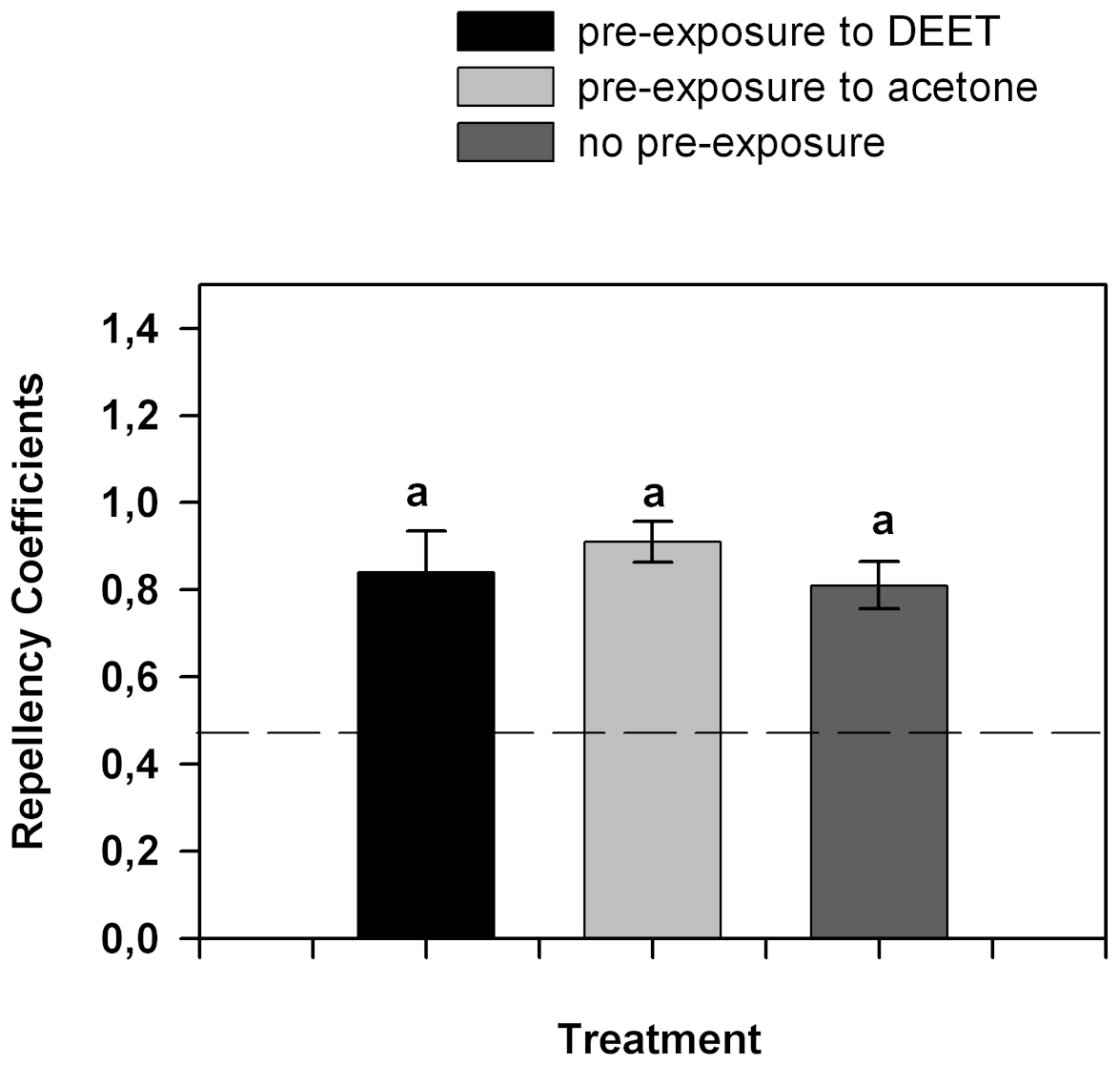
Figure 7. RC values for insects pre-exposed for 30 minutes to 700 µg/cm^2^ of DEET. Repellency was measured using a concentration of 350 µg/cm^2^ of DEET. Dotted line indicates random distribution of the insects (RC  =  0.5). Error bars indicate standard error. Equal letters indicate no significant differences (p>0.05 Kruskall-Wallis test).

Figure 7 shows the RC values for DEET (350 µg/cm^2^) in insects pre-exposed to 700 µg/cm^2^ of DEET during 30 minutes. Although the locomotor activity of insects decreased as a consequence of the pre-exposure to DEET, pre-exposed insects were equally repelled than control insects (p>0.05).

## Discussion

In this research we showed that DEET is an active repellent of the German cockroach *B. germanica*. The repellency elicited by this compound is dose-dependent. We also demonstrated that the continuous exposure to DEET decreased the locomotor activity of exposed cockroaches. In addition, we showed that the modified locomotor activity did not affect the repellency behavior caused by DEET.

The decrease of locomotor activity caused by a chemical substance may be due to an arrestant effect, or may be a symptom of intoxication of the nervous system. The arrestant effect is a reduction of insect movement as a result of undirected kinetic reactions [28]. This reduction is dose-dependent until the insect reaches the source of the arrestant compound. In nature these compounds may be semiochemicals, such as kairomones or pheromones, and may reduce or completely stop the movements of exposed insects . In *B. germanica*, cuticular hydrocarbons are the major components of their aggregation pheromone and have an attractive and arrestant activity in all the developmental stages of this insect [29]. Other authors identified alkylamines as the aggregation pheromone of *B. germanica* and obtained maximum arrestant activity with extracts of the dorsal part of the abdomen of these insects [30], [31]. On the other hand, certain compounds that are toxic to the insects’ nervous system can affect their locomotion as a symptom of poisoning.

We found in this work that insect locomotor activity does not change during exposure to DEET, so the spatial distribution of insects when repellency is tested is not biased by the effect of DEET on insect locomotion. Moreover, insects with decreased locomotor activity continued to be repelled by DEET. In addition, low concentrations of DEET were sufficient to cause repellency but were not enough to modify insect locomotor activity. Repellency and the effect on locomotor activity produced by DEET had different thresholds. Changes in locomotor activity elicited by DEET were also observed in *R. prolixus* [20]. Nymphs exposed to high concentrations of DEET showed a dose-dependent increase in their locomotor activity. Repellency occurred starting from concentrations of 70 µg/cm^2^ whereas hyperactivity was produced by concentrations higher than 700 µg/cm^2^. These results strongly suggest that the repellency response (i.e., orientation response) and the modification of the locomotor activity are non-associated phenomena. In other words, repellency is the behavioral response associated with the interaction of DEET with sensory (mainly olfactory) receptors, whereas the decrease of locomotor activity may be caused by the effect of DEET on other targets of the nervous system, possibly AChE, as recently stated by Corbel et al. [16].

We have already mentioned that insect behavior can be modified by the toxic effects of a molecule. The symptoms of insect poisoning with some neurotoxic insecticides are hyperactivity, incoordination, prostration and death [25]. If a behavioral modification observed in an insect exposed to a xenobiotic is a symptom of poisoning, the effect should continue even if the exposure has ended. In this work, we found that the decrease of the locomotor activity of cockroaches caused by DEET continued for at least 18 h after the end of the exposure, and this effect was observed after and not during exposure to the repellent. Thus, we conclude that the observed reduction in locomotor activity is most likely a toxic effect rather than a response to a sensory stimulus. Due to the total recovery of locomotor activity that was observed 24 h after exposure, a possible role of detoxifying enzymes in the target site is suggested. However, further toxicological studies are needed to confirm this hypothesis.

In summary, we demonstrated that the repellency to DEET and the modification of the locomotor activity elicited by DEET are non-associated phenomena. We suggested that the decrease of locomotor activity described in this work might be a consequence of the toxic effects caused by DEET.
